# Combination of Pembrolizumab and Stereotactic Body Radiation Therapy in Recurrent Metastatic Penile Squamous Cell Carcinoma: A Case Study

**DOI:** 10.3390/biomedicines10123033

**Published:** 2022-11-24

**Authors:** Dalia Kaakour, Steven Seyedin, Roozbeh Houshyar, Nataliya Mar

**Affiliations:** 1 Department of Medicine, Division of Hematology/Oncology, University of California, Irvine, CA 92868, USA; 2 Department of Radiation Oncology, University of California, Irvine, CA 92868, USA; 3 Department of Radiological Sciences, University of California, Irvine, CA 92868, USA

**Keywords:** penile squamous cell carcinoma, metastatic, recurrent, combination therapy, pembrolizumab, stereotactic body radiation therapy, durable response

## Abstract

The prognosis for patients with penile squamous cell carcinoma metastatic to regional lymph nodes or distant sites remains poor with limited treatment options, especially after the failure of first-line chemotherapy. Clinical trials evaluating the use of checkpoint inhibitor therapy, or the use of checkpoint inhibitor therapy with stereotactic body radiation therapy for the treatment of metastatic penile squamous cell carcinoma, are currently unavailable. In this case report, we present a patient with relapsed advanced penile squamous cell carcinoma and an unknown (human papilloma virus) HPV status and borderline programmed death-ligand 1 (PD-L)1 status who was treated with pembrolizumab and stereotactic body radiation therapy. This patient achieved a complete durable treatment response despite having genomic features of an immunologically “cold” tumor. This case highlights the importance of investigating more into the treatment of these tumors that lack genomic features that classically have been observed to be susceptible to treatment with immunotherapy or immunotherapy augmented with stereotactic body radiation therapy in solid tumors, particularly in metastatic penile squamous cell carcinoma.

## 1. Introduction

The prognosis for penile squamous cell carcinoma (pSCC), metastatic to regional lymph nodes or distant sites, remains poor, with 5-year overall survival (OS) rates of 50% and 9%, respectively [1]. The initial management of advanced pSCC involves chemotherapy, with the TIP (paclitaxel, ifosfamide, and cisplatin) regimen being preferred [2]. Although other agents have demonstrated activity in this disease, there is no preferred second-line regimen upon disease progression. As such, there is an unmet need for novel therapies in this patient population. While checkpoint inhibitors (CPIs) are routinely utilized in the treatment of various malignancies, no clinical trials evaluating their efficacy in pSCC are currently available. Several published reports have highlighted the successful use of CPIs in advanced pSCC, with some patients achieving deep and durable treatment responses [3,4,5,6,7]. The genomic profiling of these patients demonstrated features classically associated with increased responses to CPIs, including a high programmed death ligand 1 (PD-L1) expression, high tumor mutational burden (TMB), high microsatellite instability (MSI-high), and deficient mismatch repair genes (dMMRs) [3,4,6]. The concept of using stereotactic body radiation therapy (SBRT) to boost responses to CPIs in solid tumors is being investigated [8,9,10], although no data are available in pSCC. We report a case of relapsed pSCC with retroperitoneal lymph node metastasis that was treated with pembrolizumab and SBRT, achieving a complete durable treatment response, despite having genomic features of an immunologically “cold” tumor.

## 2. Case

**Figure 1:**
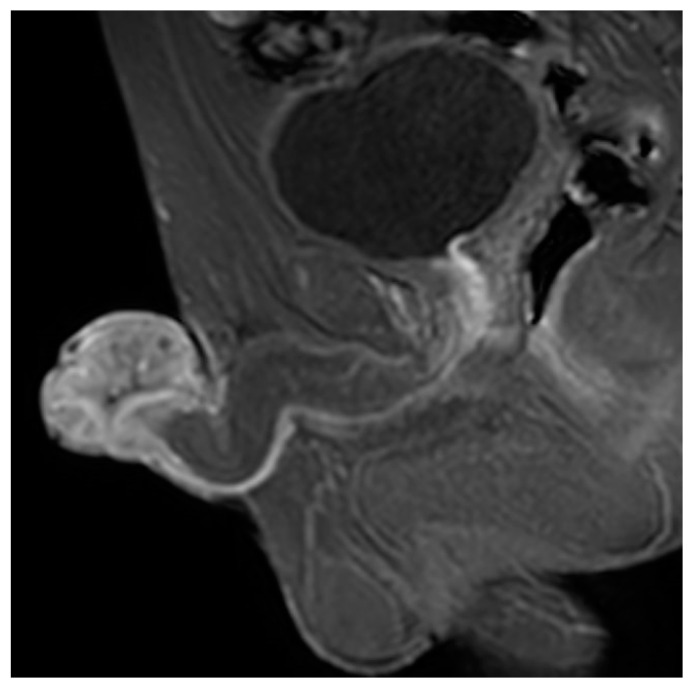
Magnetic resonance imaging (MRI) pelvis showing lobulated T2 (transverse relaxation time) intermediate mass at the level of the glans penis.

**Figure 2:**
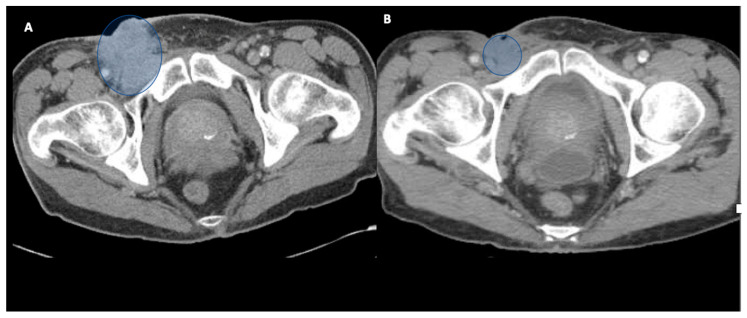
Computerized tomography (CT) scan images from baseline (**A**) and post treatment (**B**). Time points represent significant decrease in size of necrotic right inguinal nodal mass infiltrating the muscle, originally 5.3 × 5.0 cm prior to radiation therapy (RT).

**Figure 3:**
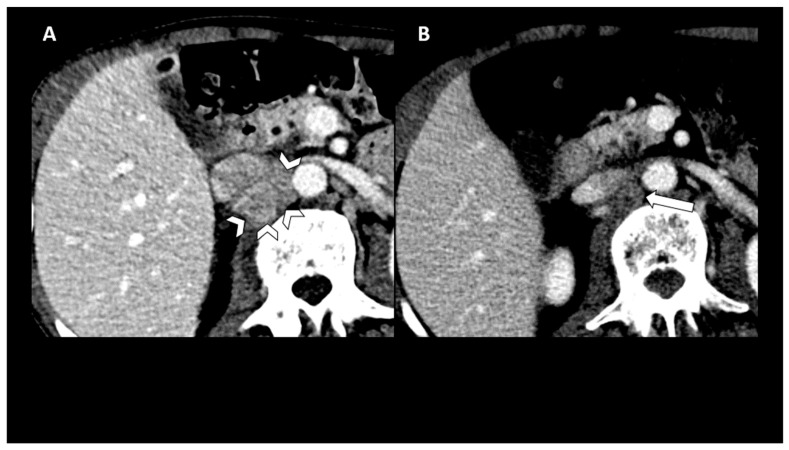
CT scan images from baseline (**A**) and post treatment (**B**). Time points represent significant decrease in size and enhancement in confluent retrocaval and aortocaval lymphadenopathy (arrowheads) with mass effect on the inferior vena cava (IVC) at baseline.

A 66-year-old man presented to an emergency department (ED) with dizziness and a progressively enlarging bleeding right groin mass over two years. Hemoglobin was 5.1 g/dl. Abdominopelvic computerized tomography (CT) scan showed a 7.5 × 6.5 × 5.6 cm mass in the right inguinal area with a skin ulceration and a 4.4 × 3.2 × 2.7 cm mass in the left inguinal area with an additional bilateral inguinal lymphadenopathy. Chest CT was negative for metastasis. A left inguinal mass biopsy revealed pSCC, although p16 staining was not performed. The patient was referred to medical oncology, but did not establish care. He presented to a different ED one month later with pain, bleeding, and drainage from the right groin mass as well as a new mass at his urethral meatus (Figure 1). A restaging CT demonstrated similar bilateral necrotic inguinal masses, new pelvic and retroperitoneal lymphadenopathy, as well as acute pulmonary emboli (Figure 1, Figure 2 and Figure 3). The patient was diagnosed with stage IV (Tx cN3 M0) penile squamous cell carcinoma.

**Figure 4:**
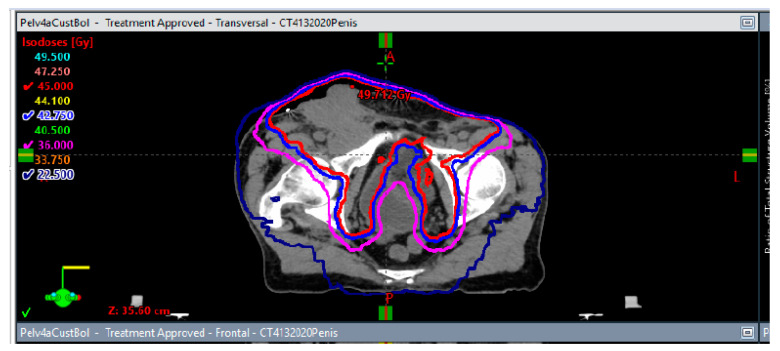

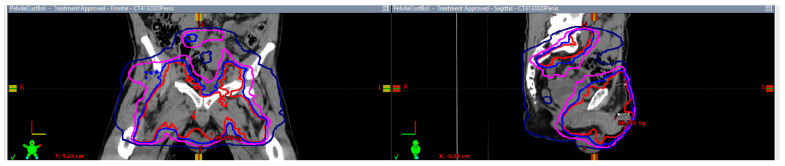
Axial, coronal, and sagittal isodose displays of initial pelvic radiotherapy plan to 45 Gy in 25 fractions. Lines in red, blue, magenta, and navy correspond with 45 Gray (Gy), 42.75 Gy, 36 Gy, and 22.5 Gy, respectively.

**Figure 5:**
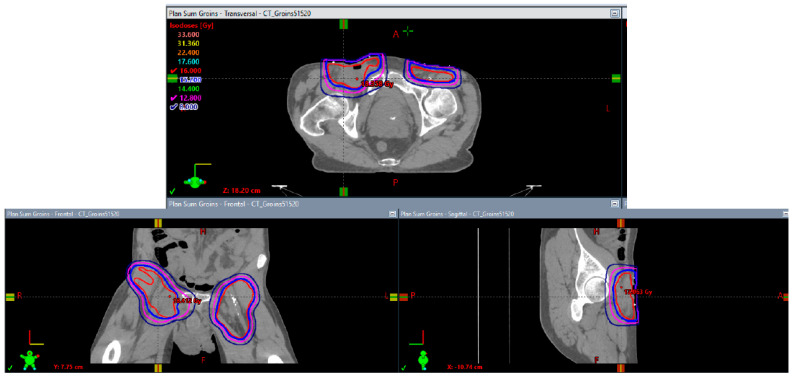
Axial, coronal, and sagittal isodose displays of inguinal groin boost plan to 16 Gy in 8 fractions. Lines in red, blue, magenta, and navy correspond with 16 Gy, 15.2 Gy, 12.8 Gy, and 8 Gy, respectively.

The patient completed six cycles of TIP chemotherapy, although paclitaxel was omitted following the first cycle due to an allergic reaction. A subsequent restaging CT revealed the resolution of the pelvic and retroperitoneal lymphadenopathy, with a significant decrease in the bilateral inguinal lymphadenopathy. However, a positron emission tomography (PET) scan performed one month later showed an increase in size and activity of the bilateral inguinal lymphadenopathy. The patient was then treated with concurrent chemoradiation with weekly cisplatin, receiving 45 Gray (Gy) in 25 fractions to the pelvic lymph nodes and a boost of 16 Gy in 8 fractions to bilateral groins and penile gross disease. A restaging PET scan after the completion of chemoradiation showed a marked decrease in the size and activity of the bilateral inguinal lymphadenopathy and the resolution of other lesions (Figure 4 and Figure 5). He then proceeded with a treatment break. Subsequent restaging imaging showed the resolution of the inguinal lymphadenopathy.

**Figure 6:**
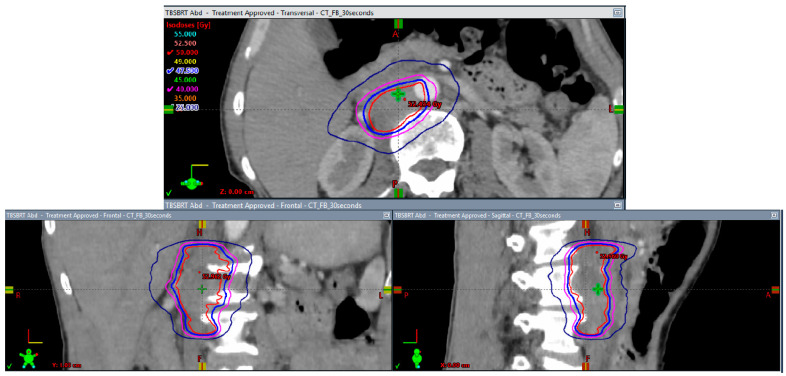
Axial, coronal, and sagittal isodose displays of the stereotactic body radiation (SBRT) plan to the aortocaval lymph node recurrence to 50 Gy in 5 fractions. Lines in red, blue, magenta, and navy correspond with 50 Gy, 47.5 Gy, 40 Gy, and 25 Gy, respectively.

A body CT ten months later showed an increase in the size of an aortocaval lymph node to 3.8 cm. Clinically, the patient developed severe back pain, requiring high doses of opiates, anorexia with weight loss of >10% of body weight, and weakness with a rapidly declining performance status. Next-generation sequencing (NGS) using Tempus on the original biopsy specimen showed a PD-L1 total positive score of 20%, TMB of 3.7 m/Mb, and microsatellite stability (MSS), as well as an RB1 p.E280* stop gain mutation, FGFR3 p.S249C missense mutation, MAPK1 p.E322K splice region variant, and KMT2D c.14383-1G>C mutation. He was initiated on pembrolizumab 400 mg every 6 weeks. Two weeks later, he proceeded with SBRT to the aortocaval lymph node, receiving 50 Gy in five fractions every other day (Figure 6). He was continued on pembrolizumab, receiving nine cycles to date. Subsequent restaging CT scans performed every 3 months demonstrated a complete treatment response (Figure 3), and he remained disease-free nearly one year post the completion of SBRT. Clinically, the patient experienced the resolution of all cancer-related symptoms.

## 3. Discussion

Penile cancers are genomically diverse, with heterogeneous signaling pathways implicated in carcinogenesis [11]. The molecular pathogenesis of pSCC can be subdivided into human papilloma virus (HPV)-dependent and -independent pathways [3]. A subset of pSCC is known to exhibit treatment responses to CPIs. PD-L1 positivity, high TMB, MSI-high, and dMMR are associated with improved treatment responses to CPIs in solid tumors [12,13,14], with pembrolizumab approved for tumor-agnostic indications of TMB ≥ 10 m/MB and MSI-high/dMMR. Although clinical outcome data in pSCC patients treated with CPIs are scarce, one report noted a dramatic clinical response to ipilimumab and nivolumab in a chemotherapy-refractory patient with high PD-L1, high TMB, MSI-high, and dMMR [3]. Another report demonstrated durable responses to pembrolizumab in two chemotherapy-refractory patients, one with high TMB and another with high PD-L1 [4]. Meanwhile, in a case series of three pSCC patients from a phase two basket trial of pembrolizumab, one patient with MSI-high experienced a durable partial response, while two other patients with MSS tumors progressed within 3 months of starting therapy [7]. These treatment outcomes in pSCC are consistent with experiences of other solid tumors harboring these genomic features. However, our patient had borderline low PD-L1, low TMB, and MSS that should have responded poorly to CPI therapy, thus, suggesting a potential synergy between pembrolizumab and SBRT.

It is well established that only a minority of patients with solid tumors respond to CPIs, partly due to varying degrees of immune surveillance [15]. Immunologically “hot” tumors contain increased levels of tumor-infiltrating lymphocytes and high neoantigen loads, making them highly recognizable by the immune system [16]. T-cell activation against tumors is determined through the complexity of the tumor microenvironment (TME), which is a milieu of tumor cells, stromal cells, suppressive cytokines, regulatory T cells, myeloid-derived suppressor cells, neoantigens, and expressed MHC molecules, as well as the PD-L1 expression of tumor cells and/or immune cells [17]. Immunologically “cold” tumors are understood to have an immunosuppressive TME and use multiple mechanisms to evade immune surveillance [18]. In pSCC, HPV-positive and HPV-negative tumors demonstrate significant differences in TME. HPV-positive pSCC is associated with higher percentages of cytotoxic CD8+ T-cells [19] and expresses lower levels of PD-L1 [20]. Meanwhile, tumor inflammatory cell infiltrates demonstrated a higher expression of Fox-P3 in HPV-negative pSCC, which is a known regulator of immune suppressive T-regulatory cells associated with unfavorable outcomes [21]. Unfortunately, p16 staining to determine the HPV status could not be performed in our case.

The effects of SBRT on the TME, including dendritic cell activation, naïve CD8+ T-cell priming, and tumor CD8+ T-cell recruitment, have been well described in multiple solid tumors [22], although no such data exist in pSCC. However, extrapolating from locally advanced head and neck squamous cell carcinoma (laHNSCC) may provide insight. Previous neoadjuvant studies with CPIs in laHNSCC showed modest treatment responses, regardless of the HPV status. For instance, major pathologic response (mPR) rates to neoadjuvant CPIs, including nivolumab and pembrolizumab monotherapy, or in combination with ipilimumab were 7–14% in HPV-negative head and neck squamous cell carcinoma (HNSCC) [23,24,25]. Meanwhile, a mPR rate of 29% was reported in HPV-positive locally advanced HNSCC patients receiving neoadjuvant durvalumab or durvalumab plus tremelimumab [26]. In contrast, a phase 1b study examined the combination of neoadjuvant SBRT at variable doses with or without nivolumab prior to definitive surgical resection in 21 HPV-positive and HPV-negative patients with laHNSCC [27]. In the entire study group, there was a 67% pathologic complete response (pCR) rate, an 86% mPR rate, and a 90% clinical to pathologic downstaging rate. Among the HPV-positive patients who received SBRT plus nivolumab, the pCR rate was 90% and the mPR rate was 100%. Among the HPV-positive patients who received SBRT alone, the pCR rate was 50%. Among all the HPV-negative patients, the pCR and mPR rates were 20% and 60%, respectively. Tissue responses were characterized by robust inflammatory infiltrates in the regression bed, plasma cells, and cholesterol clefts. It is unclear whether these results can be generalized to pSCC, but the rapid and robust treatment response in our patient was suggestive of HPV-positive disease.

The routine use of radiation therapy (RT) is controversial in pSCC [28], and treatment responses may differ between HPV-positive and HPV-negative cases. This may be due to variable genomic landscapes between these two pSCC cohorts. A retrospective study of 507 patients with pSCC who had inguinal lymph node dissection and received perioperative RT showed an improved median survival with RT in HPV-positive patients [29]. In this study, 75% of HPV-negative tumors harbored TP53 mutations versus 15% of HPV-positive tumors, which may partly explain the differences in sensitivity to RT. In another retrospective study of 51 patients, the use of chemoradiation resulted in improved 2-year locoregional control in HPV-positive patients compared to HPV-negative patients, although no genomic correlates were available in this dataset [30]. An optimal RT treatment duration, fractionation, and total dose remain controversial when used concurrently with CPIs in solid tumors, while there are no data available for pSCC. Further, the optimal timing for the administration of RT in relation to CPI therapy to optimize their synergy remains undefined. Radiation can be a double-edged sword in its impact on the immune system and can cause immunosuppression through the increased expression of regulatory T cells and the upregulation of PD-L1 on tumor cells through the production of interferon-gamma (IFN-γ) [31]. As such, more studies are needed to define the benefit and optimal delivery parameters of RT when used with CPIs, particularly in pSCC. Our case represents the first reported instance of combining SBRT with pembrolizumab in relapsed pSCC. We hypothesize that this extended duration of progression free survival was due to the immune activating synergy between ablative irradiation and CPI.

## 4. Conclusions

Penile cancers are molecularly diverse, and a subset of these tumors respond to treatment with CPIs. Predictive biomarkers, including high PD-L1, high TMB, MSI-high, and unstable MMR, which are associated with improved treatment responses to CPIs in solid tumors, may also be useful in pSCC. pSCC tumors that lack these genomic features may be characterized as immunologically “cold” and, thus, typically evade immune surveillance. The use of SBRT to boost treatment responses to CPIs in such “cold” tumors is intriguing.

## Data Availability

Not applicable.
